# Spinning together agricultural and evo-devo research for *Gynandropsis gynandra* (spider plant)

**DOI:** 10.1093/aob/mcaf205

**Published:** 2025-09-16

**Authors:** Brandi Zenchyzen, Jocelyn C Hall

**Affiliations:** Department of Biological Sciences, University of Alberta, Edmonton, Alberta T6G 2E9, Canada; Department of Biological Sciences, University of Alberta, Edmonton, Alberta T6G 2E9, Canada

**Keywords:** *Gynandropsis gynandra*, Cleomaceae, leafy vegetable, medicinal plant, opportunity crop, model organism, evo-devo, agriculture

## Abstract

**Background:**

*Gynandropsis gynandra* (Cleomaceae; formerly *Cleome gynandra*) is a leafy vegetable widely cultivated across Africa, uniquely positioned at the intersection of agricultural and evo-devo research. It is gaining recognition as an ‘opportunity crop’, valued locally for its nutritional and medicinal properties with ongoing agricultural research aimed at the development of improved cultivars and agronomic practices. Concurrently, its close evolutionary proximity to *Arabidopsis thaliana*, combined with its contrasting traits, positions *G. gynandra* as a model for studying C_4_ photosynthesis and floral development. Despite its relevance to both agricultural and evo-devo research, integration of findings between disciplines remains limited, hindered in part by inconsistent nomenclature and the lack of standardized morphological descriptors.

**Scope:**

To address this disconnect, this review synthesizes findings from agricultural and evo-devo research on *G. gynandra*. We provide an overview of its phylogenetic placement, geographical distribution, agricultural and medicinal applications, phytochemical profile, genomic and genetic resources, and morphological traits. In doing so, we emphasize the duality of *G. gynandra* as both a crop of agronomic interest and a model for evo-devo studies. Finally, we propose future research directions to promote cross-disciplinary collaboration and expedite progress in *G. gynandra* research.

**Conclusions:**

Advances in molecular tools have improved our understanding of the developmental mechanisms underlying key traits and physiological adaptations in *G. gynandra*, including C_4_ photosynthesis and antiherbivore defences. Simultaneously, morphological studies have revealed distinctive floral features and substantial phenotypic diversity, offering valuable insights for both breeding initiatives and investigations into floral development. Integrating data and resources from agricultural and evo-devo research will accelerate the improvement of *G. gynandra* and broaden its utility as a model for understanding trait evolution and development.

## INTRODUCTION

**Fig. 1.**
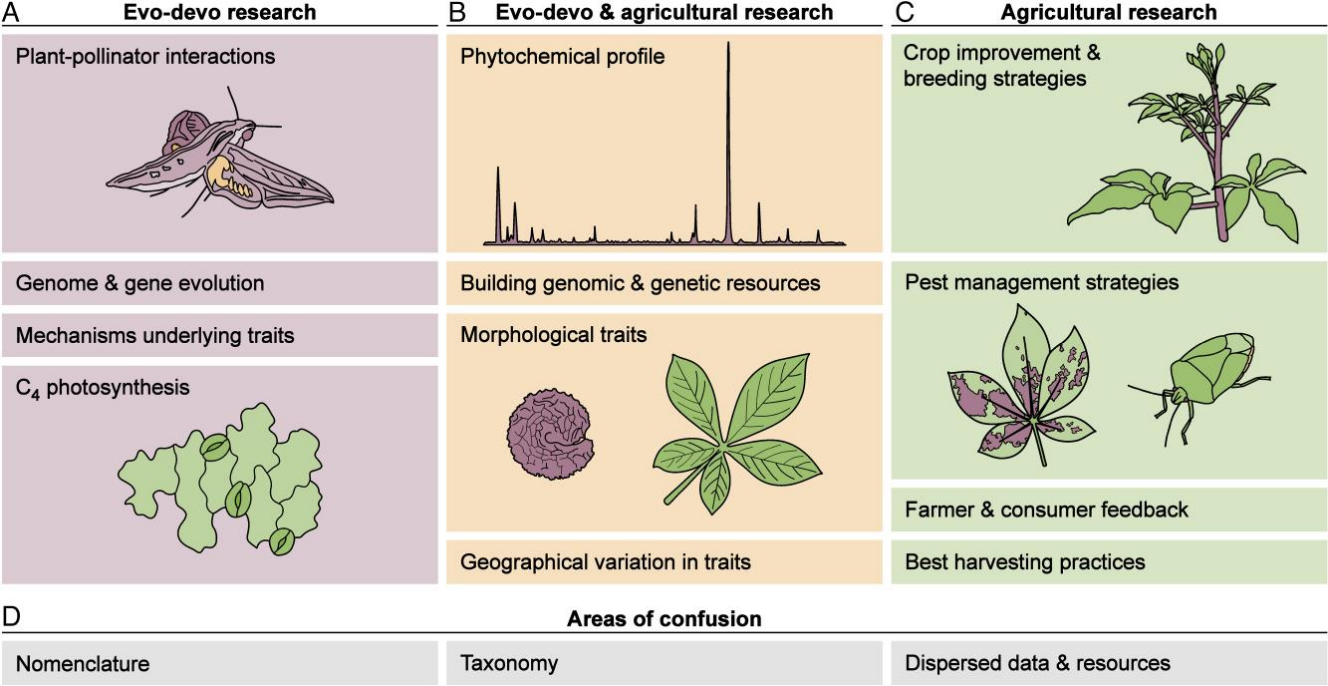
Focal areas of *Gynandropsis gynandra* research vary between disciplines. (A) Evo-devo studies centre on plant–pollinator interactions, genome and gene evolution, the mechanisms underlying traits, and C_4_ photosynthesis, while (C) agricultural studies emphasize crop improvement and breeding strategies, pest management strategies, farmer and consumer feedback, and best harvesting practices. (B) Shared resources, including phytochemical, genomic, genetic and morphological data, as well as information on geographical trait variation, offer valuable cross-disciplinary insights for both evo-devo and agricultural research. (D) However, integration is limited by inconsistent nomenclature, taxonomic confusion and scattered data and resources.

The study of *Gynandropsis gynandra* (L.) Briq. highlights the potential for cross-disciplinary insights through the integration of agricultural and evolutionary developmental biology (evo-devo) research. Yet, these fields remain largely disconnected for this flowering plant species. *Gynandropsis gynandra* (Cleomaceae), also referred to as spider plant or spider flower due to its elongated floral structures, is a nutrient-rich leafy vegetable and versatile medicinal plant native to Africa and Asia (Khuntia *et al*., 2022; Moyo and Aremu, 2022). As an essential crop for maintaining food security in resource-limited regions across Africa, *G. gynandra* is extensively studied for its agricultural-related properties such as nutrient content (Moyo *et al*., 2018; Sogbohossou *et al*., 2019; Chataika *et al*., 2021; Houdegbe *et al*., 2022*a*) and leaf yield (Houdegbe *et al*., 2018; Blalogoe *et al*., 2020; Chatara *et al*., 2023; Nzungize *et al*., 2024). Concurrently, *G. gynandra* is gaining recognition as a model organism for evo-devo research due to its close evolutionary relationship with *Arabidopsis thaliana* (Brassicaceae) and its contrasting traits including C_4_ photosynthesis (Huang *et al*., 2021; Zhao *et al*., 2023; Schreier *et al*., 2024) and atypical floral features (Zohoungbogbo *et al*., 2018; Zenchyzen *et al*., 2023*b*, 2023*a*). Despite significant advancements in each field, a disconnect persists. Agricultural reviews on *G. gynandra* often overlook potential insights from evo-devo studies, while evo-devo research is seldom linked to practical applications for crop improvement and commonly disregards data from agricultural literature. A complex taxonomic history compounds this division, with *G. gynandra* frequently referred to by its basionym *Cleome gynandra* L., outdatedly classified as a member of the family Capparaceae, or confused with other Cleomaceae species [e.g. *Tarenaya houtteana* (Schltdl.) Soares Neto & Roalson]. These inconsistencies make it challenging to determine whether journal articles are addressing the same species, further hindering cross-disciplinary research efforts (Fig. 1).

Integrated agricultural and evo-devo research offers a powerful framework to address pressing challenges in sustainable crop production while also advancing our understanding of flowering plant evolution. Agricultural research faces the urgent task of enhancing food security amid a changing climate and growing global population (Sogbohossou *et al*., 2018*a*; Henkhaus *et al*., 2020; Mashamaite *et al*., 2022). Advancements have focused predominantly on boosting the yield of a few major crop plants, resulting in just ten species dominating calorie production on cropland (Ray *et al*., 2019; Henkhaus *et al*., 2020). However, diversifying crops and domesticating underutilized species have proven effective in promoting ecosystem resilience and sustainability (Henkhaus *et al*., 2020; Knez *et al*., 2024). Meanwhile, evo-devo studies, which have long relied on model organisms, are expanding to include taxa with diverse traits (Schrager-Lavelle *et al*., 2017; Mabry *et al*., 2024; Sharma *et al*., 2024). Advances in genomic and genetic tools now enable detailed investigations into non-model species, enriching our understanding of plant evolution and diversification (Delaux *et al*., 2019). In the case of *G*. *gynandra*, morphological and phytochemical characterizations could provide critical context for evo-devo studies, while genetic discoveries have the potential to accelerate breeding and improvement programmes (Macel *et al*., 2010, Sogbohossou *et al*., 2018*a*; Jeiter and Smets, 2024). This review synthesizes findings from agricultural and evo-devo research on *G. gynandra*, providing an overview of its phylogenetic placement, geographical distribution, agricultural and medicinal uses, phytochemical profile, genomic and genetic resources, and morphological characteristics. While other reviews have focused primarily on its agronomically important traits, a comprehensive synthesis on the species’ morphology is lacking. By acknowledging key gaps and proposing future research directions, we aim to bridge agricultural and evo-devo perspectives, demonstrating their complementary potential to drive innovation and discovery in *G. gynandra* research.

## TAXONOMIC HISTORY

**Fig. 2.**
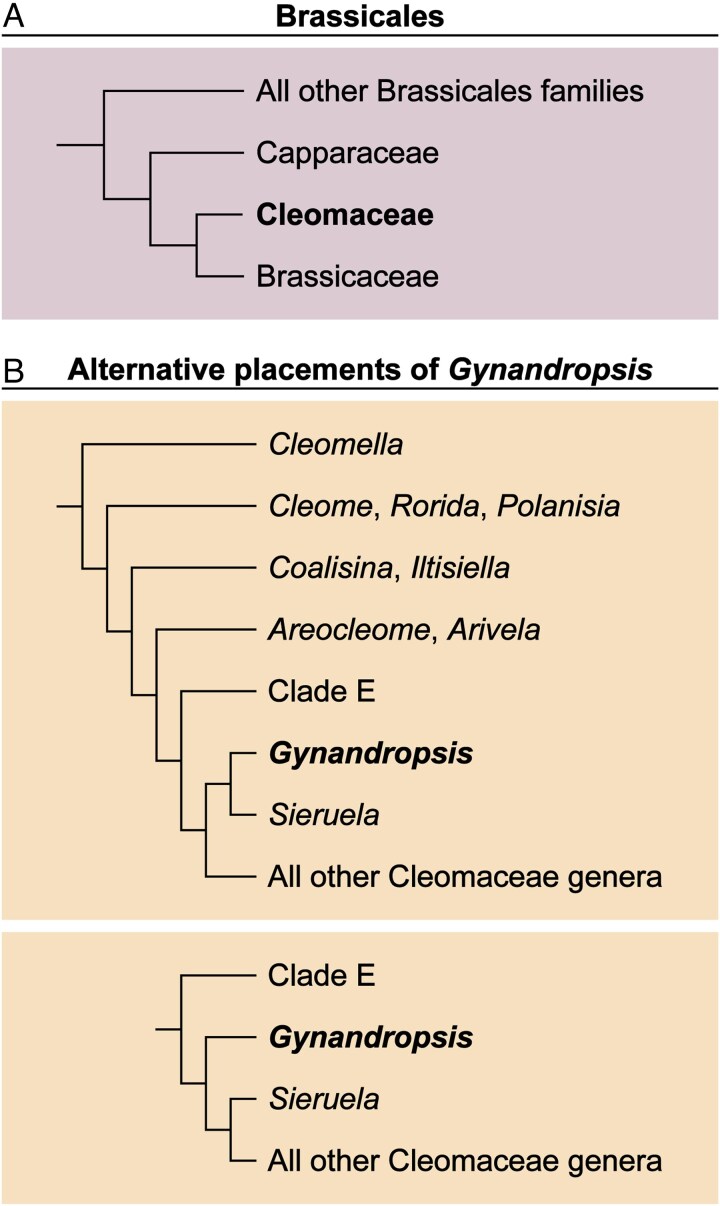
The phylogenetic placement of Cleomaceae and *Gynandropsis*. (A) Capparaceae, Cleomaceae, and Brassicaceae are separate monophyletic families within the order Brassicales. Cleomaceae is sister to Brassicaceae. Cleomaceae plus Brassicaceae are sister to Capparaceae. (B) Phylogenetic studies place *Gynandropsis* either in a monophyletic clade with *Sieruela*, or sister to *Sieruela* plus the remaining Cleomaceae genera. The Brassicales and Cleomaceae phylogenies are based on Iltis *et al*. (2011) and Saunders *et al*. (2024), respectively. Clade E: *Corynandra*, *Stylidocleome*, *Dipterygium*, *Puccionia*, *Thulinella*, *Gilgella,* and *Kersia*.

Although *G. gynandra* was previously placed within the subfamily Cleomoidae of the family Capparaceae – a classification that persists in the *G. gynandra* literature – it is now a member of the family Cleomaceae following the division of Capparaceae *s.l.* into two distinct families, Capparaceae *s.s.* and Cleomaceae (Hall *et al*., 2002; Iltis *et al*., 2011; Angiosperm Phylogeny Group *et al*., 2016) (Fig. 2A). The separation of Capparaceae *s.l.* is well supported by molecular data, which reveal three clades, Capparaceae *s.s.*, Cleomaceae, and Brassicaceae, with Cleomaceae more closely related to Brassicaceae than to Capparaceae *s.s.* (Hall *et al*., 2002; Iltis *et al*., 2011; Cardinal-McTeague *et al*., 2016; Edger *et al*., 2018 ; Hendriks *et al*., 2023). This recognition of Cleomaceae as a distinct family is further supported by morphological synapomorphies, including strongly incurved seeds, bracteate inflorescences, dry fruit with a replum (i.e. persistent placental tissue between the two valves of the capsule), and palmately compound leaves (Hall *et al*., 2002; Iltis *et al*., 2011).

Robust phylogenetic hypotheses for Cleomaceae have led to taxonomic revisions that align well-supported clades with generic classifications (Feodorova *et al*., 2010; Patchell *et al*., 2014; Roalson and Hall, 2017). Importantly, the large paraphyletic *Cleome* L. *s.l.* was segregated into multiple smaller genera (Feodorova *et al*., 2010; Patchell *et al*., 2014; Bayat *et al*., 2018). Supported by both molecular and morphological data, *G. gynandra* is the sole representative of its genus, which is more closely related to several other Cleomaceae genera (e.g. *Sieruela* Raf., *Arivela* Raf.) than it is to *Cleome s.s.* (Feodorova *et al*., 2010, Mabry *et al*., 2020; Saunders *et al*., 2024) (Fig. 2B). For simplicity, we refer to *G. gynandra* as *Gynandropsis* hereafter. The placement of *Gynandropsis* within the family remains unresolved. While some studies suggest that *Gynandropsis* is in a clade with *Sieruela*, others propose that it is sister to *Sieruela* plus several genera (Feodorova *et al*., 2010; Mabry *et al*., 2020; Saunders *et al*., 2024) (Fig. 2B).

*Gynandropsis* is a polymorphic species with a broad distribution, which has likely greatly contributed to its complex taxonomic history with more than 30 synonyms (Iltis, 1960; Roalson and Hall, 2017). Most frequently used synonyms include *Cleome gynandra*, *Cleome pentaphylla* L., and *Gynandropsis pentaphylla* L. (DC.) (Iltis, 1960). *Gynandropsis* is distinguished from other Cleomaceae genera by its three foliate bracts, white or pale coloured petals, and a prominent androgynophore and gynophore (i.e. stalk-like structures bearing the reproductive organs and pistil, respectively) (Iltis, 1960; Roalson and Hall, 2017). However, it is occasionally mistaken for other Cleomaceae taxa including the common ornamental *T*. *houtteana* (formerly *T. hassleriana*; Neto *et al*., 2022) and other species with elongated androgynophores, i.e. *Podandrogyne* Ducke and *Cleoserrata speciosa* (Raf.) Iltis. Notable morphological differences include *T. houtteana*’s stipular spines, bright pink to purple (rarely white) petals, and absence of an elongated androgynophore (Neto *et al*., 2022). *Podandrogyne* species feature unisexual flowers, arillate seeds, and often orange to red petals, while *Cleoserrata speciosa* is characterized by unifoliate bracts and pink to purple petals (seldom white) (Iltis, 1960; Iltis and Cochrane, 1989, 2007; Bayat *et al*., 2018). Additionally, *Podandrogyne* and *Cleoserrata speciosa* are native to the Americas rather than Africa and Asia (Iltis and Cochrane, 1989, 2007; Bayat *et al*., 2018; Neto *et al*., 2022).

## GEOGRAPHICAL DISTRIBUTION

*Gynandropsis* is regarded as a ‘weedy’ species, having spread extensively from Africa and Asia alongside human movements (Iltis, 1960; Shilla *et al*., 2019). It is now widespread in tropical and subtropical regions across the world, including parts of North and South America, Europe, and Australia (Iltis, 1960; Bhattacharya *et al*., 2019; Shilla *et al*., 2019). Although *Gynandropsis* is considered native to both Africa and Asia, recent biogeographical analyses predict that a *Gynandropsis* and *Sieruela* clade originated in Africa and radiated outward (Saunders *et al*., 2024). Across its species range, *Gynandropsis* exhibits extensive morphological and phytochemical variation, which is correlated with geographical origin (Sogbohossou *et al*., 2019, 2020). *Gynandropsis* thrives in a variety of soil types and environmental conditions and is commonly found near human settlements and roadsides, possibly reflecting its early introduction routes (Iltis, 1960; Bhattacharya *et al*., 2019; Shilla *et al*., 2019). The success of *Gynandropsis* in hot, sunny, and dry environments is likely due in part to C_4_ photosynthesis, an innovation that has independently evolved multiple times within Cleomaceae (Rajendrudu and Rama Das, 1982; Feodorova *et al*., 2010; Hoang *et al*., 2023). In addition to its wild populations, *Gynandropsis* is cultivated in home gardens and croplands across Africa and Asia (Iltis, 1960; Rajendrudu and Rama Das, 1982; Shilla *et al*., 2019). As a result of its broad distribution and history of human use, *Gynandropsis* has numerous vernacular names, which vary across regions (see Iltis, 1960 ; Chweya and Mnzava, 1997).

## AGRICULTURAL AND MEDICINAL USES

*Gynandropsis* is a versatile plant valued for both its agricultural and medicinal applications. Consumed primarily as a leafy vegetable, the leaves are prepared in various ways, including boiling as a potherb for use in sauces, stews, curries, and relish, pickling for a rice flavouring, and serving fresh in salads (Watt, 1890; Burkill, 1935, 1985; Chweya and Mnzava, 1997; Chataika *et al*., 2020). Tolerance for the bitterness of *Gynandropsis* leaves differs across regions (Flyman and Afolayan, 2006; Sogbohossou *et al*., 2018*a*). Methods used to reduce astringency include blanching and replacing the water prior to cooking, soaking in milk overnight, or mixing with other fruit or vegetables (Flyman and Afolayan, 2006; Onyango *et al*., 2013). Preservation practices such as blanching then sun-drying allow for year-round consumption, with dried leaves either reconstituted with water before cooking or ground and added to weaning food (Chweya and Mnzava, 1997; Flyman and Afolayan, 2006; Onyango *et al*., 2013). *Gynandropsis* seeds are crushed and made into mustard (Watt and Breyer-Brandwijk, 1932).

In addition to its culinary significance, *Gynandropsis* is occasionally used as livestock fodder (Burkill, 1985; Chataika *et al*., 2020) and has potential as a biological control by diminishing insect infestations and/or diverting insect pests from other cultivated plants such as *Brassica* crops (Brassicaceae), roses (*Rosa* hybrids; Rosaceae), and the common bean (*Phaseolus vulgaris*; Fabaceae) (Waiganjo *et al*., 2006; Nyalala and Grout, 2007; Kimbokota and Torto, 2013; Zedler *et al*., 2016). The attractiveness of *Gynandropsis* to insect pests also poses a significant challenge to its cultivation, necessitating the development of integrated pest management strategies (Francisco *et al*., 2024). Since many *Gynandropsis* farmers avoid chemical pesticides due to environmental and health risks, alternative strategies may include developing pest-resistant varieties and removing older crops, as insect infestations peak 8 weeks after sowing (Sogbohossou *et al*., 2018*a*; Francisco *et al*., 2024). Despite its susceptibility to infestations, the essential oil of *Gynandropsis* has tick repellent and acaricidal properties and has been proposed as a biological control against livestock ticks (Malonza *et al*., 1992; Lwande *et al*., 1999).

Beyond its agricultural importance, *Gynandropsis* is valued as a medicinal plant with all parts used in treating various ailments, as previously reviewed (see Khuntia *et al*., 2022; Moyo and Aremu, 2022) and summarized as follows. Leaf decoctions are traditionally used to treat malaria (Yetein *et al*., 2013), with experiments on mice supporting its efficacy (Igoli *et al*., 2016). Leaf poultices are used to alleviate pain, such as rheumatism discomfort and headaches (Burkill, 1935, 1985), as well as to treat abscesses and wounds (Shanmugam *et al*., 2012; Neamsuvan and Bunmee, 2016). *Gynandropsis* extracts possess anti-inflammatory, anti-nociceptive, and analgesic properties in rodents (Mule et al., 2008; Narendhirakannan *et al*., 2007 ; Ghogare *et al*., 2009; Bala *et al*., 2012), and anti-bacterial and anti-fungal activity (Borgio *et al*., 2008; Sridhar *et al*., 2014 ; Imanirampa and Alele, 2016 ; Kanimathi *et al*., 2019; Rotich and Mwafaida, 2024). Uses of *Gynandropsis* for pregnant women and postnatal mothers include chewing to induce labour and consuming to treat anaemia and stimulate milk production (Kamatenesi-Mugisha and Oryem-Origa, 2007; Dansi *et al*., 2008; Bosire, 2014). Clinical trials demonstrate that *Gynandropsis* consumption improves the haematological profile of lactating mothers (Bosire, 2014). The seed oil is used internally to expel parasitic worms (Watt, 1890; Burkill, 1985), and *in vitro* studies with *Gynandropsis* extracts confirm its anthelmintic properties (Ajaiyeoba *et al*., 2001; Fouche *et al*., 2016). Traditional ear-related treatments include dripping leaf sap into the ear to treat earaches and inserting the fruit into the ear canal to soften and extract wax (Watt, 1890; Watt and Breyer-Brandwijk, 1932; Burkill, 1935, 1985). It is occasionally noted that caution is needed when treating ailments with *Gynandropsis* as it may cause dermatitis (Watt, 1890; Watt and Breyer-Brandwijk, 1932; Mitchell, 1974; Burkill, 1985). *Gynandropsis* extracts also exhibit anti-cancer activity in mice (Bala *et al*., 2010). The numerous uses of *Gynandropsis* highlight the need for continued research to optimize its agronomic and pharmacological potential.

## PHYTOCHEMICAL PROFILE

Plants produce a diverse array of chemicals that are essential for regulating plant growth and development, mediating plant­–animal and plant–environment interactions, and serve as valuable nutritional and medicinal resources for human health (Hao *et al*., 2025). Metabolomics is a rapidly advancing field that has enabled comprehensive characterization of *Gynandropsis* phytochemicals through a variety of analytical methods (Sogbohossou *et al*., 2020; Hao *et al*., 2025) (Table 1). Phytochemical investigations on *Gynandropsis* have focused primarily on metabolites associated with nutrition, taste, and other health-promoting properties. Despite considerable variation in phytochemical concentrations across *Gynandropsis* accessions, the leaves are generally rich in minerals including calcium, copper, iron, magnesium, manganese, phosphorus, potassium, sodium, and zinc, which play vital roles in both plant physiology and human nutrition (Omondi *et al*., 2017; Moyo *et al*., 2018; Houdegbe *et al*., 2022*a*). *Gynandropsis* leaves are also high in vitamins, including provitamin A carotenoids (e.g. β-carotene), vitamin B (e.g. folate, riboflavin, thiamin), vitamin C (i.e. ascorbic acid), and vitamin E (e.g. tocopherols) (van Jaarsveld *et al*., 2014; Gowele *et al*., 2019; Sogbohossou *et al*., 2019). Although non-haem iron from plants is less bioavailable than haem iron found in meat, the high ascorbic acid content in *Gynandropsis* leaves may enhance iron absorption, making it a valuable dietary component for addressing iron deficiency (van Jaarsveld *et al*., 2014; Gowele *et al*., 2019; Houdegbe *et al*., 2022*a*).

**Table 1.** Phytochemical resources for *Gynandropsis* research.

Chemicals analysed	Type of tissue	Number and origin of accessions	Publication
Amino acids, fatty acids, protein	Seed	4 accessions, Zambia	Mnzava, 1990
Amino acids, minerals, fatty acids	Leaf	1 accession, Ghana	Glew *et al*., 2009
Anthocyanins, phenolics, tannins	Leaf	33 accessions, 8 African and 3 Asian countries	Chataika *et al*., 2021
Ascorbic acid, carotenoids, chlorophylls, tocopherols	Leaf	76 accessions, 4 Asian and 11 African countries	Sogbohossou *et al*., 2019
Ascorbic acid, carbohydrates, β-carotene, fatty acids, folate, minerals, protein, riboflavin, thiamin	Leaf	1 accession, South Africa	van Jaarsveld *et al*., 2014
Ascorbic acid, β-carotene, flavonoids, minerals, phenolics	Unspecified	1 accession, Zimbabwe	Moyo *et al*., 2018
Ascorbic acid, carotenoids, minerals, phytate, tocopherols	Leaf	1 accession, Tanzania	Gowele *et al*., 2019
Ascorbic acid, flavonoids, minerals, phenolics	Leaf	8 accessions, Kenya and Zambia	Jiménez-Aguilar and Grusak, 2015
β-Carotene, minerals, riboflavin	Leaf, stem	1 accession, South Africa	Schönfeldt and Pretorius, 2011
Carotenoids, flavonoids, glucosinolates, phenolics	Leaf	1 accession, Tanzania	Neugart *et al*., 2017
Fatty acids	Seed	1 accession, India	Aparadh and Karadge, 2010
Fatty acids, folic acid	Unspecified	1 accession, South Africa	van der Walt *et al*., 2009
Flavonoids	Aerial parts	1 accession, Saudi Arabia	Kasem and Fathy, 2016
Flavonoids	Stem	1 accession, Taiwan	Yang *et al*., 2008
Flavonoids, glucosinolates, minerals	Flower, fruit, leaf, stem	30 accessions, 6 African countries	Omondi *et al*., 2017
Flavonoids, phenolics	Flower, leaf and stem, seed	8 accessions, Kenya	Maina *et al*., 2021
Flavonoids, phenolics	Leaf	1 accession, India	Banoth and Thatikonda, 2019
Flavonoids, phenolics	Whole plant	1 accession, Burkina Faso	Meda *et al*., 2013
Glucosinolates	Seed	1 accession, Thailand	Songsak and Lockwood, 2002
Glucosinolates, semi-polar metabolites, volatiles	Leaf	48 accessions, 4 Asian and 10 African countries	Sogbohossou *et al*., 2020
Isothiocyanates	Seed	1 accession, unspecified	Kjær and Thomsen, 1963
Isothiocyanates	Seed	1 accession, unspecified	Kjaer *et al*., 1953
Minerals	Leaf	70 accessions, 4 Asian and 11 African countries	Houdegbe *et al*., 2022*a*
Minerals	Leaf	1 accession, Kenya	Dehayem-Kamadjeu and Okonda, 2019
Minerals	Leaf	1 accession, Kenya	Hutchinson, 2011
Minerals	Leaf	1 accession, Kenya	Orech *et al*., 2007
Minerals, phenolics	Leaf	17 accessions, 5 African countries	Thovhogi *et al*., 2021
Phenolics	Leaf	1 accession, Malaysia	Chandradevan *et al*., 2020*a*, 2020*b*
Phenolics, condensed tannins	Leaf	5 accessions, 3 African countries	Kutsukutsa *et al*., 2014
Semi-polar metabolites, volatiles	Aerial parts	1 accession, Kenya	Lwande *et al*., 1999
Terpenoids	Whole plant	1 accession, India	Das *et al*., 1999
Volatiles	Inflorescence	2 accessions, Malaysia and Malawi	Zenchyzen *et al*., 2025
Volatiles	Aerial parts	1 accession, Kenya	Kimbokota and Torto, 2013
Volatiles	Leaf	5 accessions, Kenya and Tanzania	Nyalala *et al*., 2013
Volatiles	Leaf, whole plant	5 accessions, Kenya and Tanzania	Nyalala *et al*., 2011

If the number and origin of accessions were not specified, it was assumed that a single accession was analysed and that its origin corresponded to the sampling location. Ascorbic acid: vitamin C; folate: vitamin B_9_; riboflavin: vitamin B_2_; thiamin: vitamin B_1_.

Other non-nutritive chemical classes present in *Gynandropsis* leaves include flavonoids (e.g. anthocyanins), glucosinolates, and tannins, each containing compounds with health-promoting properties such as antioxidant and anti-inflammatory activity (Omondi *et al*., 2017; Moyo *et al*., 2018; Chataika *et al*., 2021). Dietary antioxidants are important for preventing the formation and activity of reactive oxygen and nitrogen species, which can cause damage to biomolecules, such as DNA, lipids, and proteins (Halliwell, 1996). Several studies have reported high antioxidant activity of *Gynandropsis* extracts (Meda *et al*., 2013; Moyo *et al*., 2018; Chandradevan *et al*., 2020*a*; Chataika *et al*., 2021; Thovhogi *et al*., 2021). Despite their therapeutic potential, glucosinolates and tannins contribute to the bitter taste of *Gynandropsis* leaves (Songsak and Lockwood, 2002; Kutsukutsa *et al*., 2014; Chataika *et al*., 2021). The levels of astringent compounds and undesirable elements (e.g. lead, cadmium) can be altered through selective breeding as well as cooking and cultivation methods (Hutchinson, 2011; Moyo *et al*., 2016; Omondi *et al*., 2017). For example, *Gynandropsis* can accumulate more glucosinolates when grown in the field compared to a glasshouse, and both the type and amount of fertilizer can affect leaf mineral content (Hutchinson, 2011; Omondi *et al*., 2017). Achieving a balance in the concentration of astringent compounds in *Gynandropsis* leaves is key to improving its palatability while maintaining its human health benefits and ability to deter herbivores.

Although pest management is essential for cultivating healthy *Gynandropsis* plants for both agricultural and research purposes, limited studies have identified the specific compounds involved in attracting or deterring herbivorous insects (Lwande *et al*., 1999; Nyalala *et al*., 2011, 2013; Kimbokota and Torto, 2013). Specialized plant metabolites can be unpalatable, antidigestive, or toxic to herbivores, and may be constitutively produced or induced in response to herbivory (Mithöfer and Boland, 2012). Trichomes not only serve as a physical barrier but also as storage and secretion sites for these metabolites (Mithöfer and Boland, 2012; Schuurink and Tissier, 2020). Broad chemical classes involved in plant defence include glucosinolates, terpenoids, and tannins (Barbehenn and Peter Constabel, 2011; Mithöfer and Boland, 2012). In *Gynandropsis*, glucocapparin is generally the dominant glucosinolate (Kjær and Thomsen, 1963; Songsak and Lockwood, 2002; Neugart *et al*., 2017; Sogbohossou *et al*., 2020), with the highest concentration in the flowers and seeds (Omondi *et al*., 2017). When plant tissues are damaged, glucosinolates are hydrolysed into volatile sulphur-containing compounds, such as isothiocyanates (Mithöfer and Boland, 2012). Isothiocyanates have been detected in *Gynandropsis* foliar volatiles (Nyalala *et al*., 2013; Sogbohossou *et al*., 2020) and contribute to its repellency against spider mites (Nyalala *et al*., 2013).

Floral phytochemicals not only play a role in reducing florivory, but also in pollinator attraction through the production of rewards, pigments, and volatiles (Parachnowitsch and Manson, 2015). Although *Gynandropsis* secretes small amounts of floral nectar, its high sugar concentration attracts nectar foraging ants, bees, butterflies, flies, and hawkmoths (Martins and Johnson, 2013; Raju and Rani, 2016; Zenchyzen *et al*., 2024). Like several other Cleomaceae species, *Gynandropsis* nectar fluoresces vibrant blue under UV radiation (Thorp *et al*., 1975; Zenchyzen *et al*., 2024). Additional research is needed to determine the chemical basis of this fluorescence and if it serves an ecological role, such as a visual signal for pollinators, or is simply a byproduct (Thorp *et al*., 1975; Zenchyzen *et al*., 2024). Floral volatile profiles vary significantly in *Gynandropsis*, with nitrogen-containing compounds as the major component in an African accession and benzenoids predominating in an Asian accession (Zenchyzen *et al*., 2025). Similar to isothiocyanates, the nitrogen-containing floral volatiles may be degradation products or biosynthetic precursors of glucosinolates (Halkier and Gershenzon, 2006; Mithöfer and Boland, 2012). The differences in chemical profiles align with the preferences of the effective pollinators in each region­ – hawkmoths in Africa (Monteiro, 1875; Werth, 1942; Oronje, 2011; Martins and Johnson, 2013) and bees and butterflies in Asia (Burkill, 1916; Chandra *et al*., 2013; Raju and Rani, 2016). Floral volatile profiling across a broader range of accessions is needed to assess whether these patterns are consistent across African and Asian populations.

Advances in metabolomics, such as high-throughput analytical techniques and reduced costs, allows for extensive characterization of phytochemicals across plant structures and between individual plants, and in response to environmental changes (Hao *et al*., 2025). As a result, metabolomics has become a useful tool for crop improvement, facilitating the analysis of intraspecific phytochemical variation to identify nutritional targets, select parental lines for breeding programmes, and monitor changes in the progeny (Fernie and Schauer, 2009; Sogbohossou *et al*., 2018*a*; Hao *et al*., 2025). These data can also inform strategies to retain anti-herbivory compounds during cultivar development or, if such compounds compromise palatability, guide the development of botanical pesticides (Mitchell *et al*., 2016; Lengai *et al*., 2020). Similarly, metabolomics can support drug development by enabling the identification of compounds from plant extracts for biological activity screening (Shyur and Yang, 2008). Given its wide range of medicinal uses, *Gynandropsis* is a strong candidate for phytomedicine research. Finally, integrating metabolomics with genomics and transcriptomics can uncover the regulatory mechanisms and biosynthetic pathways underlying phytochemical production, offering key loci to accelerate crop improvement and insights into the evolution and diversification of specialized metabolites (Hao *et al*., 2025).

## GENOMIC AND GENETIC RESOURCES

Advancements in next-generation sequencing technologies have dramatically improved their accessibility, allowing for the research and development of non-model organisms, such as underutilized crops and taxa that possess characteristics absent in model organisms (Preston, 2021). Although work on well-established model organisms including *A. thaliana*, *Antirrhinum majus* (snapdragon; Plantaginaceae), *Oryza sativa* (rice; Poaceae), and *Zea mays* (corn; Poaceae) has significantly advanced our knowledge of plant genetics, focusing on few taxa limits our understanding of developmental networks and ability to establish a sustainable food supply as it provides a narrow representation of plant diversity (Damerval and Becker, 2017; Kamenya *et al*., 2021; Preston, 2021). Expanding genomic and genetic resources for non-model organisms is crucial for addressing fundamental questions in evo-devo (e.g. what are the molecular mechanisms underlying trait diversity?) and improving crops (Kamenya *et al*., 2021; Preston, 2021).

The order Brassicales is emerging as a model clade due to its extensive trait diversity, inclusion of *A. thaliana* along with several crops (e.g. rapeseed, capers, cruciferous vegetables, papaya, *Gynandropsis*), and rapidly expanding genomic and genetic resources (Mabry *et al*., 2024). Among these, *Gynandropsis* serves as a valuable comparative species for evo-devo research due to its contrasting traits relative to *A. thaliana* and other Cleomaceae species (Hoang *et al*., 2023; Zhao *et al*., 2023). In recent years, the germplasm collection of *Gynandropsis* has expanded significantly with the publication of two genome sequences (Hoang *et al*., 2023; Zhao *et al*., 2023) and numerous RNA sequences for a variety of plant structures, developmental stages, and experimental conditions (e.g. Zhao *et al*., 2023; Zenchyzen *et al*., 2025) (Table 2). These resources are bolstered by the close evolutionary relationship of *Gynandropsis* to *A. thaliana*, facilitating the transfer of knowledge and tools from this model species, as well as the genome sequences of Cleomaceae relatives *T. houtteana* and *Cleome violacea* L. (Cheng *et al*., 2013; Hoang *et al*., 2023; Zhao *et al*., 2023).

**Table 2.** Genomic and genetic resources for *Gynandropsis* research.

Type of data	Accession name and origin	Type of tissue	Publication	Data repository
Genome	Gyn, Malaysia	Leaf	Hoang *et al*., 2023	BioProject ID (NCBI): PRJNA843598
Genome, RNA-seq, transcriptome	Unspecified	Leaf (5 developmental stages, 2 temperature conditions), flower (2 developmental stages), capsule, stem, root	Zhao *et al*., 2023	BioProject ID (NGDC): PRJCA017363, PRJCA017364
RNA-seq, transcriptome	TOT8917, Malawi	Adaxial petals, abaxial petals, androgynophore (3 developmental stages), filaments, gynophore	Zenchyzen *et al*., 2023*a*, 2025	BioProject ID (NCBI): PRJNA680567, PRJNA1030768
RNA-seq, transcriptome	Taiwan	Leaf (developing)	Huang *et al*., 2017, 2021	BioProject ID (NCBI): PRJNA714768, PRJNA381122
RNA-seq	Unspecified	Leaf (6 developmental stages), seedling (3 developmental stages), seed (3 developmental stages), sepal, petal, stamen, carpel, stem, root	Külahoglu *et al*., 2014	BioProject ID (NCBI): PRJNA237449
RNA-seq	Unspecified	Leaf (3 regions), leaf mesophyll cells, leaf bundle sheath cells	Aubry *et al*., 2014*a*	BioProject ID (NCBI): PRJNA243610
RNA-seq	Unspecified	Leaf mesophyll cells, leaf guard cells	Aubry *et al*., 2016	ArrayExpress (EBI): E-MTAB-3379
miRNA-seq	Unspecified	Leaf (2 developmental stages)	Gao *et al*., 2017	BioProject ID (NCBI): PRJNA377356
Targeted DNA sequence (Angiosperms353)	857511.0 (PRE), South Africa	Leaf	Saunders *et al*., 2024	BioProject ID (NCBI): PRJNA1080677
RNA-seq, DNAseI-seq	Malaysia-01, Malaysia	Seedling (5 developmental stages/light treatments)	Singh *et al*., 2024	BioProject ID (NCBI): PRJNA640984
DNaseI-seq	Unspecified	Leaf (3 developmental stages/light treatments)	Reyna-Llorens *et al*., 2018	BioProject ID (NCBI): PRJNA419285
RNA-seq	Unspecified	Seedling (2 developmental stages/light treatments)	Burgess *et al*., 2016	ArrayExpress (EBI): E-MTAB-4355
Genome survey sequence, RNA-seq	Unspecified	Leaf	Mabry *et al*., 2020	BioProject ID (NCBI): PRJNA542714
Chloroplast genome sequence	DS20200516011 (NF), China	Leaf	Shi *et al*., 2021	BioProject ID (NCBI): PRJNA687638
QTL	TOT7200, Malaysia × TOT8917, Malawi; TOT7199, Malaysia × TOT8918, Malawi	Leaf	Simpson *et al*., 2025	GitHub: plycs5/GgQTL

NCBI: National Center for Biotechnology Information; NGDC: National Genomics Data Center; EBI: European Bioinformatics Institute; PRE: South African National Biodiversity Institute’s National Herbarium; NF: Herbarium of Nanjing Forestry University.

Both shared and independent polyploidy events have shaped the evolutionary history of Brassicaceae and Cleomaceae, including a whole-genome duplication (*Gg*–α) within Cleomaceae that is shared by *Gynandropsis* and *T. houtteana* (Mabry *et al*., 2020; Hoang *et al*., 2023). Genome and gene duplications may serve as drivers of evolutionary change, giving rise to novel traits through subfunctionalization and neofunctionalization, where duplicated genes either partition ancestral functions or acquire entirely new roles (Birchler and Yang, 2022). These events are associated with the origin of methionine-derived glucosinolates in the Cleomaceae, Brassicaceae, and Capparaceae clade – a chemical defence that diversified through an evolutionary arms race with butterflies (Edger *et al*., 2015; van den Bergh *et al*., 2016). Additionally, duplications have played a role in the independent evolution of C_4_ photosynthesis in at least three Cleomaceae species (Voznesenskaya *et al*., 2007; Koteyeva *et al*., 2011; van den Bergh *et al*., 2014; Huang *et al*., 2021; Hoang *et al*., 2023). While *Gynandropsis* has been extensively studied for its C_4_ photosynthesis mechanism (reviewed in Schlüter and Weber, 2020; Huang *et al*., 2023), the broader Brassicales order offers a comparative framework for understanding the evolution of different photosynthetic strategies (Marshall *et al*., 2007; Bayat *et al*., 2018; Mabry *et al*., 2024).

Though genomic and genetic resources are invaluable for identifying candidate genes, functional analyses are essential for confirming their roles. To facilitate such studies, a transformation system has been developed for *Gynandropsis* (Newell *et al*., 2010), and has since been used in investigations of the mechanisms underlying C_4_ photosynthesis (Huang *et al*., 2013; Aubry *et al*., 2014*b*; Williams *et al*., 2016; Reyna-Llorens *et al*., 2018). While stable transformation enables permanent gene edits that can be used for crop improvement, selective breeding remains a vital and cost-effective approach – especially for the often-underfunded advancement of ‘orphan crops’ (Kamenya *et al*., 2021; Venezia and Creasey Krainer, 2021). The development of effective breeding strategies benefits from knowledge on trait heritability and genetic variation (Kangai Munene *et al*., 2018; Sogbohossou *et al*., 2018*a*; Simpson *et al*., 2025). In *Gynandropsis*, several agronomically important traits, including days to flowering, plant height, and leaf biomass, have shown moderate to high broad-sense heritability and genetic advance, suggesting that selection based on phenotype is likely to produce measurable improvements in these traits over successive generations (Kiebre *et al*., 2017; Kangai Munene *et al*., 2018; Chatara *et al*., 2023; Mativavarira *et al*., 2024*b*; Simpson *et al*., 2025). Furthermore, quantitative trait loci (QTL) associated with key agronomic traits have been identified, providing the foundation for marker-assisted selection of parental lines (Simpson *et al*., 2025). Ultimately, integrating these genomic, genetic, and functional resources with accurate morphological descriptions and thorough chemical characterizations is essential for unravelling the mechanisms underlying trait evolution (Endress, 2002; Jeiter and Smets, 2024). This holistic approach could be utilized for crop improvement by informing the development of breeding strategies and genome editing protocols that optimize favourable traits (e.g. high leaf yield, vitamin content) (Sogbohossou *et al*., 2018*a*; Kamenya *et al*., 2021; Simpson *et al*., 2025).

## MORPHOLOGICAL TRAITS

Morphological data continue to play a vital role in the molecular era of biology (Endress, 2002; Jeiter and Smets, 2024). Not only are detailed morphological studies essential for accurate species identification, but they can also provide critical insights into the form and function of plant structures (Endress, 2002; Jeiter and Smets, 2024). Although individual studies tend to focus on few features (e.g. only vegetative or only floral), data from both agricultural and evo-devo research, including several early in-depth anatomical studies, provide a cohesive picture of *Gynandropsis* morphology (e.g. Raghavan, 1939; Murty, 1953; Houdegbe *et al*., 2022*b*; Zenchyzen *et al*., 2025). *Gynandropsis* is an erect annual herb, reaching up to 2m in height, with variable branching (unbranched to profusely branched, bearing numerous inflorescences) (Iltis, 1960; Wu *et al*., 2018; Sogbohossou *et al*., 2019; Parma *et al*., 2023). The leaves are palmately compound with three to seven leaflets (typically 5-foliate), petiolate, estipulate, and alternately arranged (Iltis, 1960; Puri, 1971; Tucker and Vanderpool, 2010; Das *et al*., 2022). Both stems and petioles vary in vestiture (glabrous to glandular-pilose) and in colour (green to purple) (Iltis, 1960; Masuka *et al*., 2012; Wasonga *et al*., 2015; Wu *et al*., 2018; Sogbohossou *et al*., 2019). Leaflets are glabrous to pubescent and range in shape from oblanceolate to elliptic or rhombic, acuminate to acute (less often rounded) at the apex, and attenuate to cuneate at the base, with entire to serrulate or denticulate margins (Iltis, 1960; Puri, 1971; Tucker and Vanderpool, 2010; Wu *et al*., 2018; Sogbohossou *et al*., 2019; Das *et al*., 2022). The leaves have anatomical features associated with C_4_ photosynthesis including Kranz anatomy, characterized by a concentric arrangement of bundle sheath and mesophyll cells around vascular bundles (i.e. veins), large bundle sheath cells, few mesophyll cells between veins, and high vein density (Voznesenskaya *et al*., 2007; Koteyeva *et al*., 2011; Bayat *et al*., 2018). Along with the above-mentioned variation in plant form, there is considerable range in the size of vegetative and floral structures (Iltis, 1960; Wu *et al*., 2018; Sogbohossou *et al*., 2019; Houdegbe *et al*., 2022*b*).

**Fig. 3.**
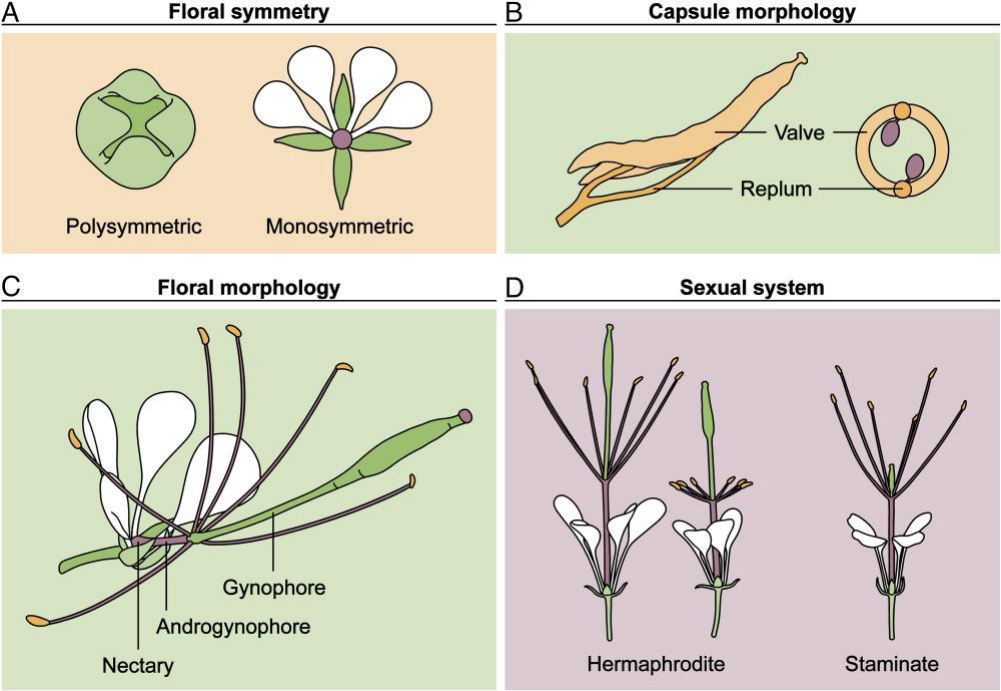
Key floral features and developmental traits of *Gynandropsis gynandra*. (A) The flower transitions from polysymmetric early in development to monosymmetric at anthesis, (B) has a pistil that matures into a capsule with valves that dehisce from the replum to release the seeds, (C) has notable structures including a receptacular nectary and an elongated androgynophore and gynophore, and (D) has an andromonoecious sexual system with staminate flowers and two types of hermaphroditic flowers, one with fully developed stamens and another with sessile stamens.

The inflorescences are terminal racemes with numerous flowers and trifoliate bracts located at the base of each pedicel (Iltis, 1960; Zohoungbogbo *et al*., 2018). The flowers typically have four sepals (distinct, green, lanceolate to obovate), four petals (distinct, white or pale coloured varying from yellow to pink or purple, unguiculate with an obovate to suborbicular blade), a receptacular nectary, an elongated androgynophore and gynophore, six stamens (purple filaments), and a bicarpellate pistil (short style, capitate stigma) (Raghavan, 1939; Iltis, 1960; Tucker and Vanderpool, 2010; Das *et al*., 2022; Zenchyzen *et al*., 2023*b*). Flowers are monosymmetric (i.e. bilaterally symmetric/zygomorphic) at maturity due primarily to the upward curvature of the petals and stamens (Patchell *et al*., 2011) (Fig. 3A). However, during early organogenesis, the floral buds are polysymmetric (i.e. actinomorphic), aside from a slightly enlarged abaxial sepal (Patchell *et al*., 2011). Monosymmetry is established in the late stages of development when the stamen filaments are elongating and protruding adaxially out of the closed petals (Patchell *et al*., 2011; Zenchyzen *et al*., 2023*a*). The fertilized pistils of *Gynandropsis* flowers develop into dry, linear-cylindrical capsules (Iltis, 1960; Iltis *et al*., 2011), often referred to as pods (e.g. Zohoungbogbo *et al*., 2018; Wu *et al*., 2018) (Fig. 3B). The two valves of the capsule dehisce from the replum to disperse the seeds (Iltis *et al*., 2011). Unlike siliques, the characteristic fruit type of Brassicaceae, the capsules of Cleomaceae species, including *Gynandropsis*, lack a false septum (Hall *et al*., 2002; Iltis *et al*., 2011). Seeds are reniform to subglobose (i.e. strongly incurved with a narrow open cleft) with a rugulose to tuberculate and dark brown or black seed coat (Iltis, 1960; Iltis *et al*., 2011; Kwarteng *et al*., 2018; Blalogoe *et al*., 2020). While several studies provide detailed descriptions of the floral development, internal anatomy, and external morphology of *Gynandropsis* (Raghavan, 1938, 1939; Murty, 1953; Iltis, 1960; Patchell *et al*., 2011; Zohoungbogbo *et al*., 2018; Parma *et al*., 2023; Zenchyzen *et al*., 2023*b*, 2023*a*), we focus on floral traits associated with pollination and herbivory, as these are relevant to both agricultural and evo-devo research.

Floral structures of *Gynandropsis* that likely facilitate pollinator interactions include the petals, androgynophore, gynophore, and nectary (Fig. 3C). The androgynophore and gynophore presumably increase the likelihood of cross-pollination by optimally positioning the reproductive organs for pollinator contact and/or reduce the chance of self-pollination by spatially separating the stigma and anthers (Medan and Ponessa, 2003; Shakarishvili and Osishvili, 2013; Scorza and Dornelas, 2014; Rocha *et al*., 2015; Zenchyzen *et al*., 2023*a*). For instance, hawk moths with short proboscises feeding of the nectar near the base of the androgynophore contact the elevated reproductive organs while hovering over the flower (Oronje, 2011; Martins and Johnson, 2013). Although both stalk-like structures probably contribute to pollination, the androgynophore of *Gynandropsis* has been more thoroughly studied than the gynophore. The androgynophore is a relatively uniform cylindrical structure that rapidly elongates primarily through cell expansion during the late stages of floral development (Zenchyzen *et al*., 2023*a*). It contains a vascular cylinder, which diverges near the apex of the androgynophore into an outer ring of vascular bundles supplying the filaments and an inner ring that extends through the length of the gynophore (Raghavan, 1939; Murty, 1953; Zenchyzen *et al*., 2023*a*). This vascular pattern differs from the androgynophore of *Cleoserrata speciosa*, where the staminal bundles remain independent from the vascular cylinder throughout its entire length (Iltis, 1960). The receptacular nectary of *Gynandropsis* has an annular ring of nectary parenchyma (Murty, 1953; Zenchyzen *et al*., 2023*b*). Nectar is secreted via nectarostomata, often forming four droplets opposite the sepals (Raju and Rani, 2016; Zenchyzen *et al*., 2023*b*).

Micromorphology of the floral epidermis can influence pollinator interactions as well as provide defence against florivory and other environmental stressors (Riglet *et al*., 2021). In *Gynandropsis*, the epidermal cells of the petals, stamen filaments, and anthers are decorated with intricate striations (Zenchyzen *et al*., 2025). The cellular surfaces of the petal blades and anthers have disordered striations, while the petal claws and distal portion of the filaments (near the anthers) have linear (i.e. ordered) striations (Zenchyzen *et al*., 2025). Striations can generate structural colour through angle-dependent scattering of light, resulting in iridescence or blue halos (Moyroud *et al*., 2017). These optical effects may contribute to pollinator attraction by enhancing the flower’s visual signals (Moyroud *et al*., 2017; Riglet *et al*., 2021). In addition to striations, papillate epidermal cells are found on the petal claws, stigma, and sometimes the nectary of *Gynandropsis* flowers (Zenchyzen *et al*., 2025). Papillae may contribute to the visual signals and tactile cues of the flower by enhancing UV-absorption/-reflection patterns and improving grip for pollinators (Whitney *et al*., 2009; Alcorn *et al*., 2012; Schulte *et al*., 2019). Further, papillae can reduce the wettability of the floral structures, potentially mitigating adverse effects of surface wetness on plant–pollinator interactions, such as reduced light reflection and increased susceptibility to pathogen infection (Taneda *et al*., 2015). The epidermal surface of *Gynandropsis* flowers also includes glandular trichomes, which typically produce and store secondary metabolites (Schuurink and Tissier, 2020; Zenchyzen *et al*., 2025). In *Gynandropsis*, trichomes are located on the abaxial surface of the sepals and the valves of the pistil, presumably contributing to the defence of developing flowers and seeds against florivory (Zenchyzen *et al*., 2025).

The sexual system of *Gynandropsis* has been described as both polygamomonoecious and andromonecious, characterized by the presence of staminate, pistillate, and hermaphrodite flowers, or staminate and hermaphrodite flowers, occurring on the same plant, respectively (Zohoungbogbo *et al*., 2018; Parma *et al*., 2023) (Fig. 3D). The hermaphrodite flowers have a functional ovary and fertile stamens but vary in gynophore length, while the staminate flowers have a short or absent gynophore and a residual ovary (Raghavan, 1939; Murty, 1953; Raju and Rani, 2016; Zohoungbogbo *et al*., 2018; Parma *et al*., 2023). Flowers with sessile stamens have been described both as pistillate and hermaphroditic (Raju and Rani, 2016; Parma *et al*., 2023). However, Raju and Rani (2016) reported pollen production in flowers with sessile stamens, suggesting that andromonoecious is the most accurate term to describe the sexual system of *Gynandropsis*. In addition to the various reproductive morphs, several abnormal floral morphs have been documented (Raghavan, 1939; Murty, 1953). These include flowers with an extra sepal or petal, adnation of the petals to the androgynophore or the stamens to the gynophore and pistil, petaloid sepals, sepaloid or staminoid petals, an open ovary with exposed ovules, or a tri- or tetracarpellate pistil (Raghavan, 1939; Murty, 1953). Further research on the diverse and abundant floral morphs of *Gynandropsis* could be used to address questions regarding floral development and its underlying mechanisms (Meyerowitz *et al*., 1989), while understanding its sexual morphs is relevant for developing effective breeding programmes (Zohoungbogbo *et al*., 2018).

## OUTSTANDING QUESTIONS AND FUTURE DIRECTIONS

### Comprehensive sampling for comparative studies

Comparative studies have revealed geographical patterns in *Gynandropsis* phenology, C_4_ photosynthesis, morphology, phytochemistry, and pollinators, with variation between Asian, west African, and southern/east African accessions (Reeves *et al*., 2018; Wu *et al*., 2018; Sogbohossou *et al*., 2019, 2020; Chataika *et al*., 2021; Houdegbe *et al*., 2022*a*, 2022*b*; Zenchyzen *et al*., 2025). These patterns suggest that there may be genetically distinct, geographically separate subspecies of *Gynandropsis*, a hypothesis that could be supported with additional comparative studies using comprehensive geographical sampling. For example, genome sequences of west and southern/east African accessions would complement the existing Asian accession genome to provide insight into genomic differences and a more comprehensive picture of trait evolution and diversification. Integrated breeding programmes could utilize such knowledge while incorporating stakeholder input to define breeding objectives and develop improved cultivars (Sogbohossou *et al*., 2018*a*). Comparative studies play a crucial role in this process by identifying accessions with desirable traits for targeted breeding strategies (Chataika *et al*., 2021; Houdegbe *et al*., 2022*a*). Given the geographical differences, it is crucial for both agricultural and evo-devo research to specify the origin and accession of *Gynandropsis* plants used in studies to ensure the accurate interpretation and broader applicability of findings.

### Alternative functional study approaches

Understanding the regulatory networks underlying traits is a fundamental component of evo-devo research and essential for developing efficient and effective breeding programmes (Sogbohossou *et al*., 2018*a*; Delaux *et al*., 2019; Kamenya *et al*., 2021). However, in *Gynandropsis*, transformation is the only established functional genomic tool for validating gene function. An alternative approach that circumvents the time-intensive development of stable transformants and allows for targeted suppression of candidate genes is virus-induced gene silencing (VIGS) (Purkayastha and Dasgupta, 2009). *Cleome violacea* has an established VIGS protocol, which has been used to study floral nectary initiation (Carey *et al*., 2021, 2023); however, its effectiveness in *G. gynandra* has yet to be evaluated. Expanding the functional genomic toolkit by testing and optimizing the *Cleome violacea* VIGS protocol for *Gynandropsis* would provide a valuable alternative for gene function studies. A key advantage to having an established transformation protocol is that it serves as a prerequisite for CRISPR/Cas, a powerful genome editing tool capable of generating stable and heritable mutations (Venezia and Creasey Krainer, 2021). Additionally, the availability of *Gynandropsis* genome sequences, along with the well-annotated genome of its close relative *A. thaliana*, facilitates CRISPR/Cas applications by enabling precise genomic target selection (Kamenya *et al*., 2021; Venezia and Creasey Krainer, 2021). The integration of CRISPR/Cas into *Gynandropsis* research would significantly accelerate the development of improved cultivars (Kamenya *et al*., 2021; Venezia and Creasey Krainer, 2021). To the best of our knowledge, *Gynandropsis* genome editing by CRISPR/Cas has not been documented in the scientific literature.

### Building community across disciplines

Fostering collaboration across agricultural and evo-devo disciplines is key to accelerating research and maximizing the potential of *Gynandropsis*. Stakeholder surveys involving farmers and consumers (e.g. Sogbohossou *et al*., 2018*b*; Chataika *et al*., 2020; Francisco *et al*., 2024; Mativavarira *et al*., 2024*a*; Stoll *et al*., 2025) not only guide crop improvement strategies but also inform evo-devo research by identifying agriculturally relevant traits that might otherwise be overlooked. To ensure that findings are transferable, documenting the specific *Gynandropsis* accession studied and its seed source is key, especially given the species’ substantial phenotypic and genetic diversity. While examining a wide range of accessions is valuable for understanding intraspecific variation, focusing on accessions prioritized in breeding programmes or those with a reference genome can provide particularly meaningful insights. At the same time, consistent use of terminology and up-to-date taxonomy is critical for improving the discoverability of relevant literature. Although the term ‘orphan crop’ is commonly used to describe *Gynandropsis* and other underutilized species, it may not be the most appropriate due to its social connotations and inconsistent definitions (Lara *et al*., 2023). Alternatively, the term ‘opportunity crop’ offers a more positive and inclusive framing, highlighting the value and untapped potential of locally significant yet unimproved species (Simpson *et al*., 2025). *Gynandropsis* expands on this concept, not only offering opportunities for agricultural advancement but also serving as a focal species in evo-devo research – enhancing our understanding of complex processes such as C_4_ photosynthesis and floral development.

## CONCLUSIONS

In this review, we have highlighted key areas of interest and ongoing research in agricultural and evo-devo contexts, including polyploidy, functional studies, C_4_ photosynthesis, floral morphology, sexual systems, and secondary metabolites. By integrating these shared interests with expanding genomic and genetic resources, morphological and phytochemical characterization, and documented within-species variation, *Gynandropsis* is well poised for synthetic investigations in crop improvement as well as plant development and evolution. To fully utilize this potential, the cohesive use of nomenclature will facilitate clearer communication, resource sharing, and data integration. Overall, improved collaboration across disciplines will accelerate the development of *Gynandropsis* as a model for both applied and fundamental plant science.
